# Unraveling Flow Patterns through Nonlinear Manifold Learning

**DOI:** 10.1371/journal.pone.0091131

**Published:** 2014-03-10

**Authors:** Flavia Tauro, Salvatore Grimaldi, Maurizio Porfiri

**Affiliations:** 1 Department of Mechanical and Aerospace Engineering, New York University Polytechnic School of Engineering, Brooklyn, New York, United States of America; 2 Dipartimento di Ingegneria Civile, Edile e Ambientale, Sapienza University of Rome, Rome, Italy; 3 Dipartimento per l’Innovazione nei Sistemi Biologici, Agroalimentari e Forestali, University of Tuscia, Viterbo, Italy; NASA Jet Propulsion Laboratory, United States of America

## Abstract

From climatology to biofluidics, the characterization of complex flows relies on computationally expensive kinematic and kinetic measurements. In addition, such big data are difficult to handle in real time, thereby hampering advancements in the area of flow control and distributed sensing. Here, we propose a novel framework for unsupervised characterization of flow patterns through nonlinear manifold learning. Specifically, we apply the isometric feature mapping (Isomap) to experimental video data of the wake past a circular cylinder from steady to turbulent flows. Without direct velocity measurements, we show that manifold topology is intrinsically related to flow regime and that Isomap global coordinates can unravel salient flow features.

## Introduction

The characterization of complex flows is a major challenge in climatology, biology, and engineering [1], [2], [3], [4]. The detection of salient flow features is traditionally addressed through the analysis of velocity fields, obtained from flow visualization, numerical, and analytical methodologies [5], [6], [7], [8], [9], [10]. Specifically, flows are classified by estimating relevant physical parameters [11], [12], [13], through pattern tracking procedures [14], [15] or flow topology analysis [16], [17], [18]. These approaches rely on the availability of computationally expensive measurements to accurately describe the flow field. Beyond flow characterization, an even more elusive problem in fluid mechanics is the real time control of flow structures in biology, biomedicine, aerodynamics, and environmental science [19], [20]. Despite recent technological advances, such as the use of microelectromechanical systems and the introduction of feedback control [21], [22], flow manipulation is still affected by limitations in measuring relevant flow parameters, data storage, and computational time [23]. These drawbacks hamper real time autonomous flow monitoring of complex systems.

Here, we propose the implementation of a machine learning framework for unsupervised characterization of fluid flows. Different from established flow visualization techniques that require a-posteriori intensive processing of high resolution images [24], [25], our approach uses raw video data to rapidly disclose and examine relevant flow phenomena. Moving forward from pattern tracking, machine learning demonstrates remarkable potential in identifying features underlying complex phenomena [26], [27]. Specifically, manifold learning aims at uncovering the low dimensional structures “hidden” in high dimensional data. For instance, the isometric feature mapping (Isomap) embeds large scale data sets on lower dimensional manifolds approximated by undirected graphs, whose topology is utilized to compute geodesics on the true nonlinear manifolds [28]. This machine learning algorithm focuses on the extraction of relevant features directly from images without requiring the intermediate phase of quantitative parameters estimation [29]. In particular, the Isomap algorithm is effectively applied to the problem of face and human motion recognition [30] and collective behavior in biological systems [31], [32], [33] supporting the feasibility of using Isomap in fluid dynamics.

To demonstrate our approach, we study the flow past a circular cylinder by processing flow visualization video data with Isomap for Reynolds numbers ranging from 50 to 1725. For such range, the fluid experiences steady separation, the formation of regular vortex patterns (that is, von Karman vortex streets), and the initiation of turbulence. We anticipate Isomap to detect flow regimes through varying dimensionality of the embedding manifolds, similarly to the problem of collective behavior of animal groups, where dimensionality is showed to relate with the degree of coordination between individuals [31], [32], [33]. The flow around a circular cylinder is widely studied in the literature [34], [35], [36], [37], [38] for its numerous instances in nature [39] and engineering [40]. In our study, this phenomenon is instrumental to experiment with an array of different flow regimes, spanning from steady to periodic and unsteady. We design an experimental setup including a hollow circular cylinder of outer diameter *D* positioned vertically at the cross-section of a water tunnel. A dye-injection system is developed for improved visualization of the flow streaklines around the cylinder through a digital camera (see the Methods for further details). We vary the flow regime by changing the free stream velocity, *U*.

In the framework of nonlinear machine learning, we regard experimental video frames as the Isomap ambient space and seek to characterize the flow by studying the embedding manifolds. We demonstrate that the topology of the embeddings can be associated with the flow regime, whereby lack of flow separation is manifested through one dimensional manifolds and the presence of coherent structures through higher dimensionality. Further, we show that manifold inspection can be used to estimate the frequency of vortex shedding and study flow pattern variations due to externally-induced perturbations.

## Results

### Flow Separation Correlates with Embedding Dimensionality

**Figure 1:**
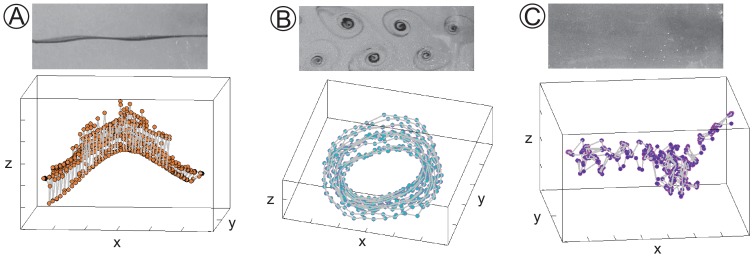
Enhanced contrast pictures and three dimensional embedding manifolds for three different experimental data sets. Images are reported for (A) *Re* = 50, (B) *Re* = 159, and (C) *Re* = 1725.

We process experimental video data recorded with a commercial camcorder with the Isomap algorithm and study the relationship between the topological features of the embedding manifolds and the flow regime, controlled by the Reynolds number *Re* (see the Materials and Methods for the full set of *Re* adopted in the experiments). The Reynolds number is defined as 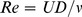, where 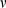 is the kinematic viscosity of water (at the measured fluid temperature of 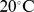). In line with our expectations, we find that data relative to steady flow separation, that is, 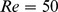, are embedded onto one dimensional manifolds, see Figure 1(a). Conversely, for 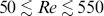, that is, for flow regimes characterized by a transition from laminar to turbulent von Karman vortex streets [34], cylindrical manifolds are obtained, see Figure 1(b). From 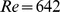, when turbulent flow coexists with periodic fluctuations in the cylinder wake [41], larger amounts of data points are not embedded onto cylindrical surfaces and rather fall onto irregularly shaped manifolds that are well approximated by nearly one dimensional structures, see Figure 1(c).

### Manifold Global Coordinates Unravel Flow Features of Von Karman Vortex Streets

**Figure 2:**
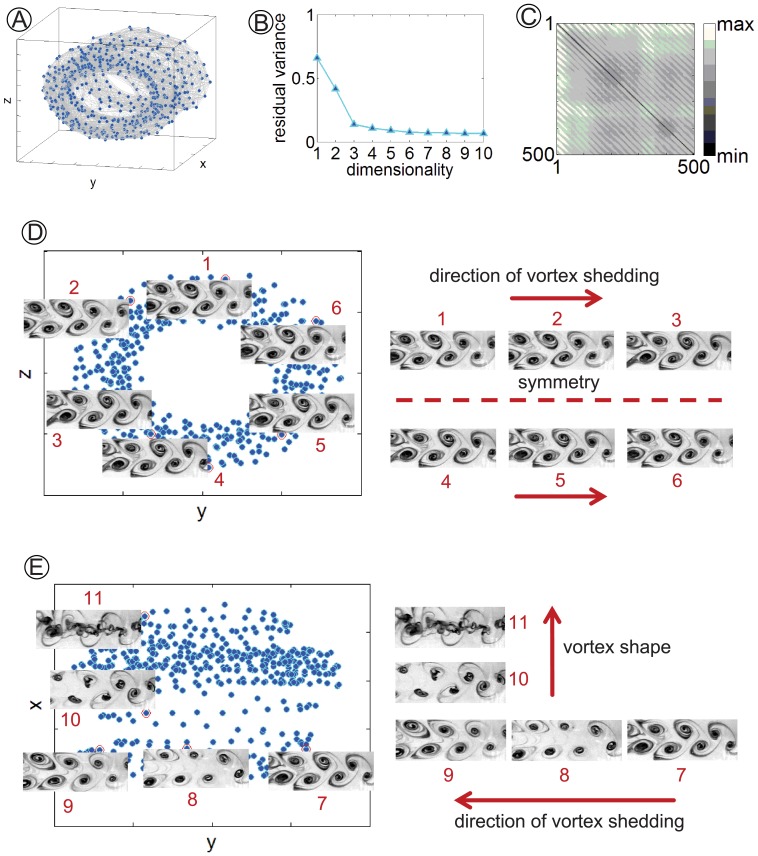
Manifold global coordinates for *Re* = 191. (A) Three dimensional representation of the embedding manifold. (B) Residual variance of the data set against dimensionality; values are reported up to dimensionality equal to 10. (C) Distance matrix for the data set as computed by Isomap. (D) Two dimensional projection on the *yz*-plane of (A); images 1 to 6 correspond to selected data points on the annulus. (E) Two dimensional projection on the *xy*-plane of (A); images 7 to 11 correspond to selected data points on the embedding (Contrast and brightness in video frames are enhanced for readability).

Figures 2(a–c) display the cylindrical manifold, residual variance, and distance matrix obtained by setting 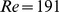. We find that data points are arranged onto a thick cylindrical structure; specifically, 90% of the data set is represented by a three dimensional manifold (see the residual variance for dimensionality equal to three). Further, the distance matrix highlights the periodicity of the flow through the presence of regular sets of points that are closer to their neighbors (see the diagonal stripes in Figure 2(c)).

We further find that the topology of the embedding is related to two major features underlying the experimental data set. Specifically, in the two dimensional projection in Figure 2(d), all data points are symmetrically distributed along an annulus, suggesting a periodic behavior. By counterclockwise inspection of the annulus, we observe that data are consecutively ordered along the flow direction. Moreover, data points located at similar angular positions tend to depict comparable shapes. Variations along the thickness of the cylinder, corresponding to its radial coordinate, are related to varying image contrast during the experiment. Diametrically opposed locations on the annulus show vortex shedding phases that differ by 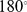. Thus, one of the Isomap global coordinates, corresponding to the angular coordinate along the cylinder mantle, identifies the periodicity of the observed flow. Projecting the three dimensional embedding on a plane parallel to its axis, we find that images are horizontally ordered in the direction of flow, Figure 2(e). Further, variations of the flow pattern in the data set are arranged along the vertical direction, corresponding to the axial coordinate of the cylinder, with images displaying differently shaped vortices arranged far apart on the manifold.

### The Topology of the Embedding Manifolds can be Used to Estimate Salient Flow Parameters

**Figure 3:**
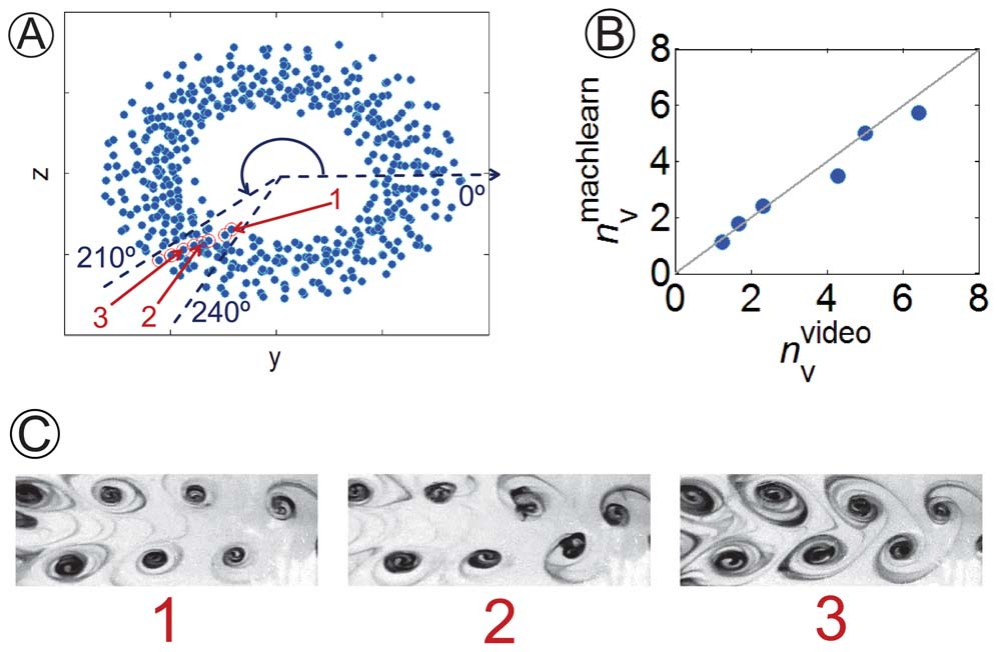
Vortex shedding frequency estimation for 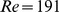. (A) Two dimensional projection on the 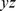-plane of the embedding manifold (blue dots correspond to experimental data points and red circled markers are video frames laying at a comparable angular position on the annulus. Images 1 to 3 are selected video frames used for vortex shedding frequency estimation. All of them depict similar vortex patterns. Shedding frequency is computed by dividing the number of coherent structures shed from image 1 to 2 (and 2 to 3) by the respective time interval. Contrast and brightness in video frames are enhanced for readability). (B) Comparison of vortex shedding frequency obtained from the procedure illustrated in (A), 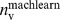, to values computed from vortex counting, 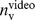 (the solid line is the bisectrix).

We quantify the vortex shedding frequency by inspection of the annular projections recovered for 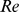 from 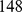 to 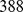. Specifically, we manually compute from the video feed the number of vortices, 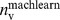, formed between images laying at comparable angular positions on the annulus, see Figure 3(a) for the randomly selected sector between 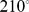 and 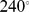. Further, we compare our results to estimations obtained by counting in the video feed the number of vortices shed in known time intervals. For the sector of the cylinder in Figure 3(a), computed values, 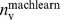, are consistent with findings from vortex counting, 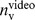, see Figure 3(b) (root mean squared error, RMSE, equal to 0.45 with respect to the bisectrix).

### Data Cluster Differently on Manifolds of Varying Dimensions as a Function of the Flow Parameters

**Figure 4:**
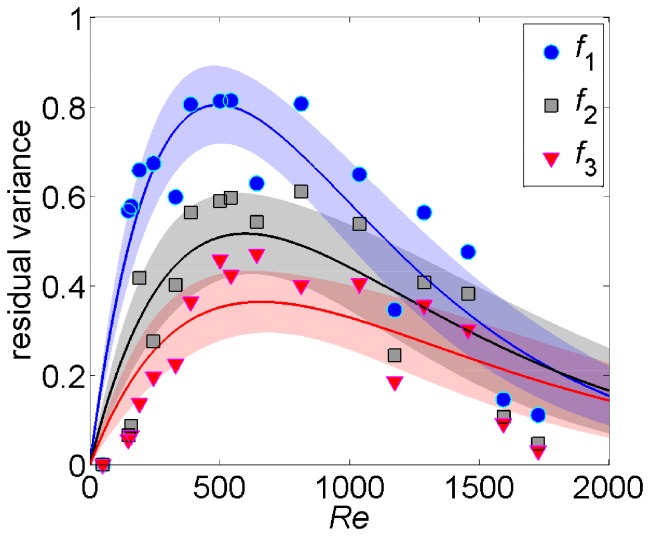
Residual variance against different flow regimes. Markers correspond to residual variances for the first three embedding dimensionalities (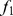, 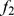, and 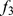 for dimensionality 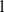, 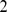, and 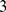, respectively). Blue, black, and red solid lines are best-fit curves (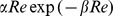) for dimensionality equal to one, two, and three, respectively. Shaded areas correspond to 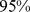 confidence intervals).

Our analysis of the dimensionality of Isomap embeddings demonstrates a close correspondence between the algorithm outputs and the flow physics. We further elucidate such relations by studying the residual variances for the first three dimensionalities of the data sets, which capture the vast majority of the experiments (more than 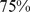 of the data). In Figure 4(a), we present residual variances for all the experimental data sets fitted by functions of the form 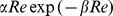, with 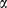 and 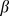 being unknown parameters (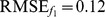, 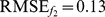, and 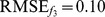), where shaded regions denote the 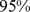 confidence intervals. As expected, we find that at low and high *Re*, the flow can be described through nearly one dimensional embeddings, which capture the translational motion in the video feed. On the other hand, as coherent structures are shed by the cylinder, data points are fit on higher dimensionality manifolds, which also account for the shape of the vortices. We observe that increasing the degree of turbulence of the flow corresponds to “hiding” periodic fluctuations in the flow. Indeed, Isomap captures the prevalently translational nature of the data.

## Discussion

In this study, we present an unsupervised approach for characterizing flow patterns based on isometric feature mapping. The methodology does not rely on computationally expensive pattern tracking procedures or on the analysis of flow velocity fields [14], [15], [18]. Rather, it requires minimal preprocessing of experimental video frames (see the Materials and Methods for details).

Our results show that the dimensionality of the embedding manifold and its topology are landmarks of the flow regime, whereby smooth one dimensional manifolds are constructed from steady flows, cylindrical embeddings from von Karman vortex streets, and irregular structures from turbulent flows. With respect to von Karman vortex streets, our results are in agreement with the analysis presented in [17], where proper orthogonal decomposition is conducted on particle image velocimetry (PIV) and analytical velocity fields for flow characterization. In fact, we obtain striped distance matrices and two dimensional annular embedding projections for vortex shedding similar to [17]. This is achieved by directly processing video images through Isomap rather than performing computationally expensive PIV. Notably, we recover such annular projection also when the Isomap input space is constituted of unordered sets of experimental video frames, suggesting that our procedure can be successfully used to independently sort the ambient space in time.

In line with our expectations, we also find that Isomap global coordinates of the embedding manifolds relate with relevant features of the flow. For example, the axial coordinate of the cylinder in Figure 2(e) captures variations in vortex shape and provides a measure of the wake regularity. These variations in the geometry of the shed vortices are well studied in fluid dynamics [42] and can be related to flow-induced vibrations of the cylinder, boundary-layer effects, and inhomogeneities in the free stream velocity field. Although speculative, our findings also suggest that the method can be used to estimate pertinent flow parameters by exploiting the nonlinear dependence of the residual variance on the flow parameters. Specifically, the analysis of the residual variances associated with the first few embedding dimensionalities can be leveraged to extract usable information for the identification of flow parameters.

Raw video feed is also considered in [43] to study flow kinematics. Therein, images are obtained from a PIV study and the optical flow technique is utilized to reconstruct the velocity field. Here, we rely on standard video feed for rapid unsupervised characterization of flow phenomena through global features. While the accuracy of optical flow techniques is highly dependent on image quality and tracer seeding uniformity in the field of view, Isomap emphasizes underlying flow characteristics through relative topological distance among video data points, thus reducing the effect of fixed pattern noise in the images.

In contrast to canonical vortex detection methodologies [6], [8], [12], [14], no preprocessing in terms of scaling, compression, or filtering is performed on images before nonlinear embedding through Isomap. Nonetheless, the performance of the methodology relies on the visibility of the flow structures and, therefore, low contrast, poor resolution, and highly nonuniform background noise may require image enhancement before feature extraction. While not explored in this study, such image enhancement can be achieved through computationally inexpensive and automated procedures that are commonly executed in flow visualization applications [25]. Ultimately, we emphasize that increasing the size of the dataset is expected to improve on the estimations of Isomap geodesic distances (see the Methods for details), and, therefore, aid the identification of embedding manifolds.

Our results indicate that unsupervised nonlinear machine learning through the Isomap algorithm can be successfully used to rapidly unravel salient flow features. Real time flow monitoring is a major challenge when image-based methodologies are needed rather than invasive sensors and probes. For instance, we expect this methodology to find application in biofluidics, where flow characterization can aid in monitoring hemodynamics, oxygen transport, intravascular blood pressure, and blood vessel obstructions [44], [45], [46], [47], [48]. Further, unsupervised flow characterization is anticipated to provide insight in environmental sensing, where noninvasive methodologies are increasingly needed for monitoring the evolution of large scale natural systems [39], [49], [50]. In addition, the approach may find application in autonomous robotics for rapid environmental mapping of unknown areas [51].

## Materials and Methods

### Experimental Setup

Experiments are conducted in an open-test section water tunnel (Engineering Laboratory Design 502S). The tunnel cross-section is 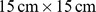. Along the water flume, a working cross-section is selected at approximately 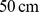 in between two honeycomb grids for improved uniformity of the velocity profile. A hollow copper cylinder of outer diameter equal to 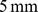 is positioned vertically in the center of the working cross-section. Two 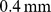 injection ports located at the mid-span of the cylinder at an angle of 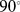 from the front stagnation point allow for homogeneous and continuous rhodamine WT injection in the flow through a syringe system. Dye streaklines are captured by a Canon Vixia HG20 digital video camera, located 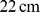 underneath the water tunnel and 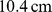 downstream the working cross-section, with its axis perpendicular to the plane of vortex shedding. The camcorder acquires a field of view equal to 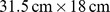; its resolution is set to Full HD (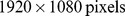); and its acquisition frequency is kept at 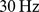. Experiments are performed for Reynolds numbers equal to 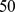; 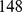; 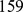; 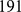; 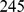; 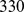; 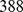; 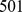; 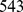; 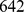; 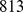; 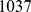; 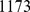; 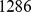; 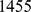; 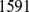; 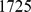. Different flow regimes are generated by varying the free stream velocity in the tunnel. This is achieved by adjusting the flume motor frequency from 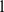 to 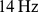, corresponding to an average flow velocity varying from approximately 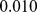 to 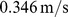 at the mid-span of the working cross-section as per an independent PIV analysis.

### Isomap Algorithm

The Isomap algorithm is a nonlinear manifold learning methodology for dimensionality reduction problems [27]. Differently from the classical multidimensional scaling method (MDS), Isomap uses geodesic rather than Euclidean manifold distances between data points. The algorithm objectives are: i) embedding a data set of 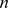 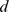-dimensional data points on a manifold, ii) defining the manifold dimensionality, and iii) finding such dimension to be much less than 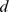. In particular, for the data set 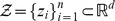, Isomap constructs a corresponding data set 
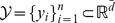
 and assesses if 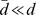. The 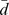-dimensional embedding is represented through the parametrization 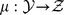, where each 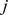-th coordinate of the 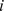-th data point is parameterized as 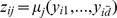, for 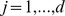, and for each data point 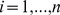. The second subscript is used to identify vector components. The algorithm follows these steps [28], [31], [32], [33]:

1. *Construction of the neighbor graph* 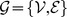 *to approximate the manifold*. The elements of the set of vertices 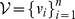 match the data points 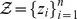 and the elements of the set of edges 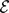 are unordered pairs of vertices in 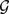. Edges connect 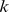-nearest neighbors vertices. Specifically, edges 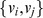 correspond to the 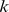-closest data points 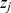 to 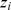, for each 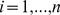, with respect to the Euclidean distance in the ambient space (the pixels space), denoted by 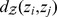. The matrix 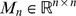, encoding the weighted graph of intrinsic manifold distances corresponding to 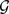, is computed. For each 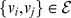, the distance equals the 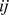-th entry of 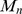, that is, 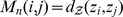. For all 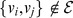, 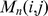 is set equal to 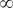 to prevent jumps between branches of the underlying embedding.
2. *Computation of the graph geodesic matrix* 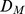 *to approximate the geodesic of the manifold.* Floyd's algorithm [52] is utilized to compute shortest paths. From 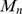, an approximate geodesic distance matrix 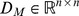 is computed, whose 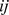-th entry is the shortest path length from 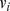 to 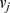, being an approximation of manifold geodesic distances.
3. *Approximation of the manifold distance by* 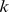-*nearest neighbor distance*. The matrix 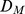 computed in the previous step is used to approximate the geodesic distances of the manifold between 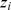 and 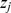 by the graph distance between 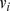 and 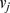. If the data density is too low, a poor representation of the manifold could be obtained with some neighbors lying on separate manifold branches.
4. *Computation of the projective variables* 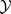 *applying the classical MDS on the matrix* 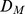. Classical MDS [53] is performed on a matrix of dissimilarities between pairs of input and candidate embeddings, which minimize the distance in the embedded manifold. For a survey on MDS, see [31].

The outputs of Isomap are the transformed data points on an embedding manifold for the input data set 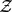 and the vector 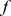 of residual variances, which represents the fraction of data points not embedded on the manifold for different dimensions.

### Video Data

Experimental videos are decompressed into “.jpg” image files and sequences of 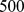 consecutive frames are selected for manifold learning. Such sequences are retained by performing a preliminary test where the homogeneity in image intensity is assayed and sets of images with marked differences in coloration discarded. This test is conducted to prevent the algorithm from relating data dimensionality to nonhomogeneities in dye injection. Before processing, images are cropped around the plane of vortex shedding to display a field of view of 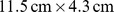 corresponding to 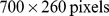. Only the red channel (where pixel intensity varies from 0 to 255) is extracted for Isomap processing. For each flow regime, Isomap is applied to data sets comprising 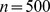 arrays of 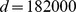 dimensional data points, where each array corresponds to a reshaped raw image. The nearest neighbors parameter is set to 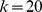 based on similarity among consecutive images. To test the stability of the methodology, the Isomap algorithm is rerun on subsets of subsampled images and varying the value of 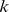. We find that embedding manifold topologies are consistently recovered for values of 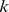 ranging from 15 to 25 for the same data set.

### Residual Variances Fitting

The vectors of the residual variances for the first three embedding dimensionalities are plotted against the respective 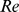 for each experimental video. Such data points are fitted through the nonlinear least squares method with functions of the type 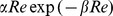, where 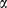 and 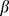 are fitting parameters. The 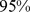 confidence intervals are estimated based on the fitting model coefficient covariance matrix.

### Vortex Shedding Frequency

Vortex shedding frequency is evaluated for experiments conducted at 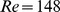; 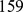; 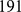; 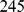; 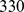; and 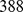. For such data sets, the frequency obtained from images located at comparable angular positions on the annular embedding projection is compared to vortex shedding frequencies estimated through the analysis of randomly selected sets of 10 to 40 consecutive images of the same videos. Similar to [54], frequencies are found by counting the number of vortices convected past a selected reference point in consecutive pictures in known time intervals. The duration of the time intervals is computed from the camera acquisition frequency.
